# Supplementary figures and images for: Essential role of an ERV-derived Env38 protein in adaptive humoral immunity against an exogenous SVCV infection in a zebrafish model
Source: PLoS Pathog. 2023 Apr 4;19(4):e1011222. doi: 10.1371/journal.ppat.1011222 (PMC10072453; doi:10.1371/journal.ppat.1011222)

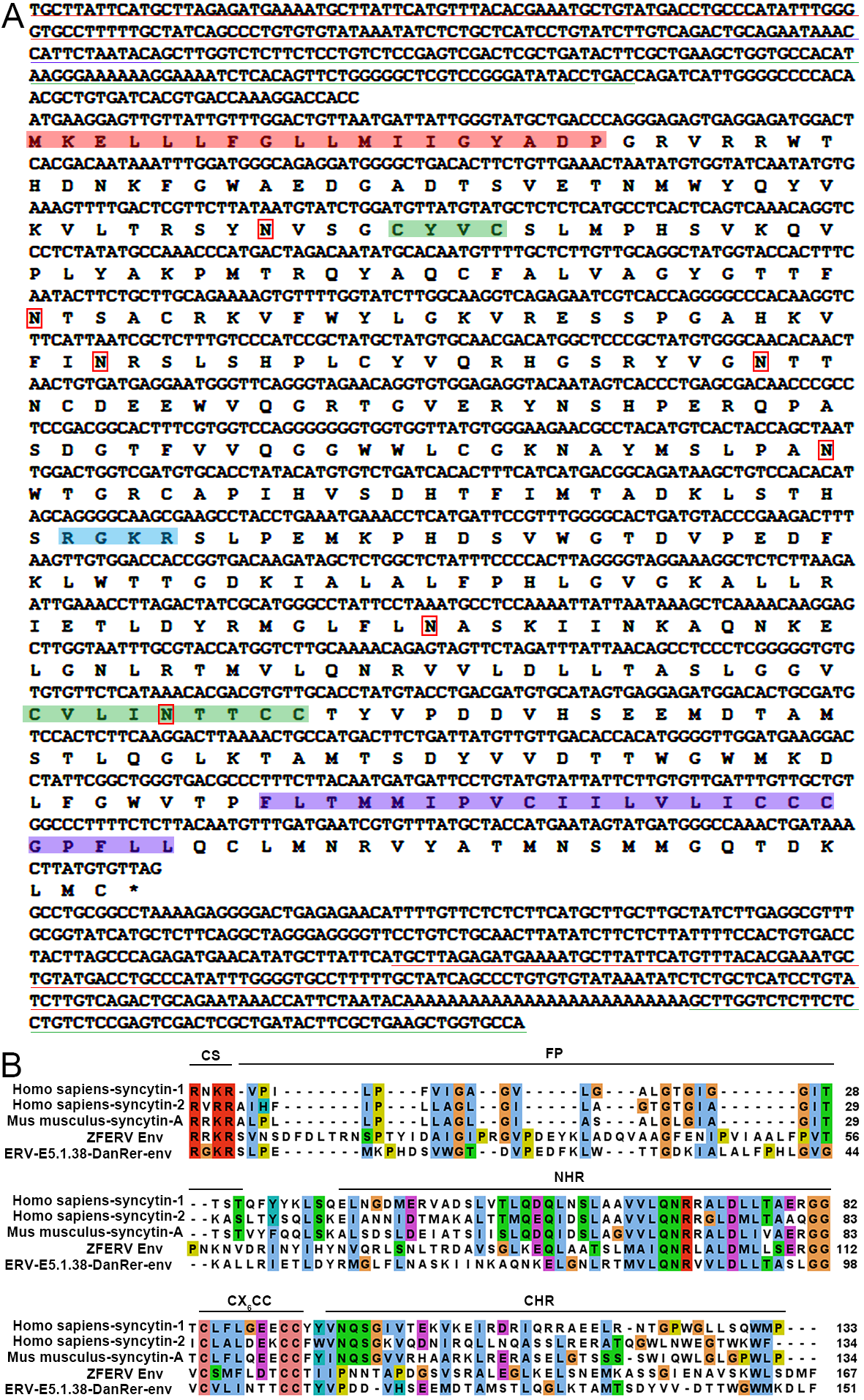

Supplement: S1 Fig — (A) The cDNA sequence of env38 gene and the deduced amino acids of Env38 protein. The non-coding regions represent the LTR, in which the U3, R and U5 sequences were underlined with the red line, purple line and green line, respectively. The signal peptide and transmembrane domain of the Env38 protein were bottomed with red and purple, respectively. The sequences forming conserved disulfide bonds were bottomed with green. The potential SU-TM cleavage site was bottomed with blue. Seven aspartic acids forming deduced N-glycosylation sites in Env38 protein were framed with red. The asterisk represented the stop codon. (B) Multiple alignment of the amino acid sequence of Env38 with a previously described ZFERV Env protein of zebrafish and Syncytin proteins of mammals. The relatively conserved motifs or amino acids in the protease cleavage site (CS), the fusion peptide (FP), the N-terminal heptad repeats (NHR), the C-terminal heptad repeats (CHR) and the linker motif between NHR and CHR (CX6CC) were marked with different colors. The Genbank accession numbers of the sequences were as follows: Homo sapiens-Syncytin-1, NP_001124397.1; Homo sapiens-Syncytin-2, NP_997465.1; Mus musculus-Syncytin-A, NP_001013773.1; ZFERV Env, AAM34209.1. (TIF) [file ppat.1011222.s002.tif]

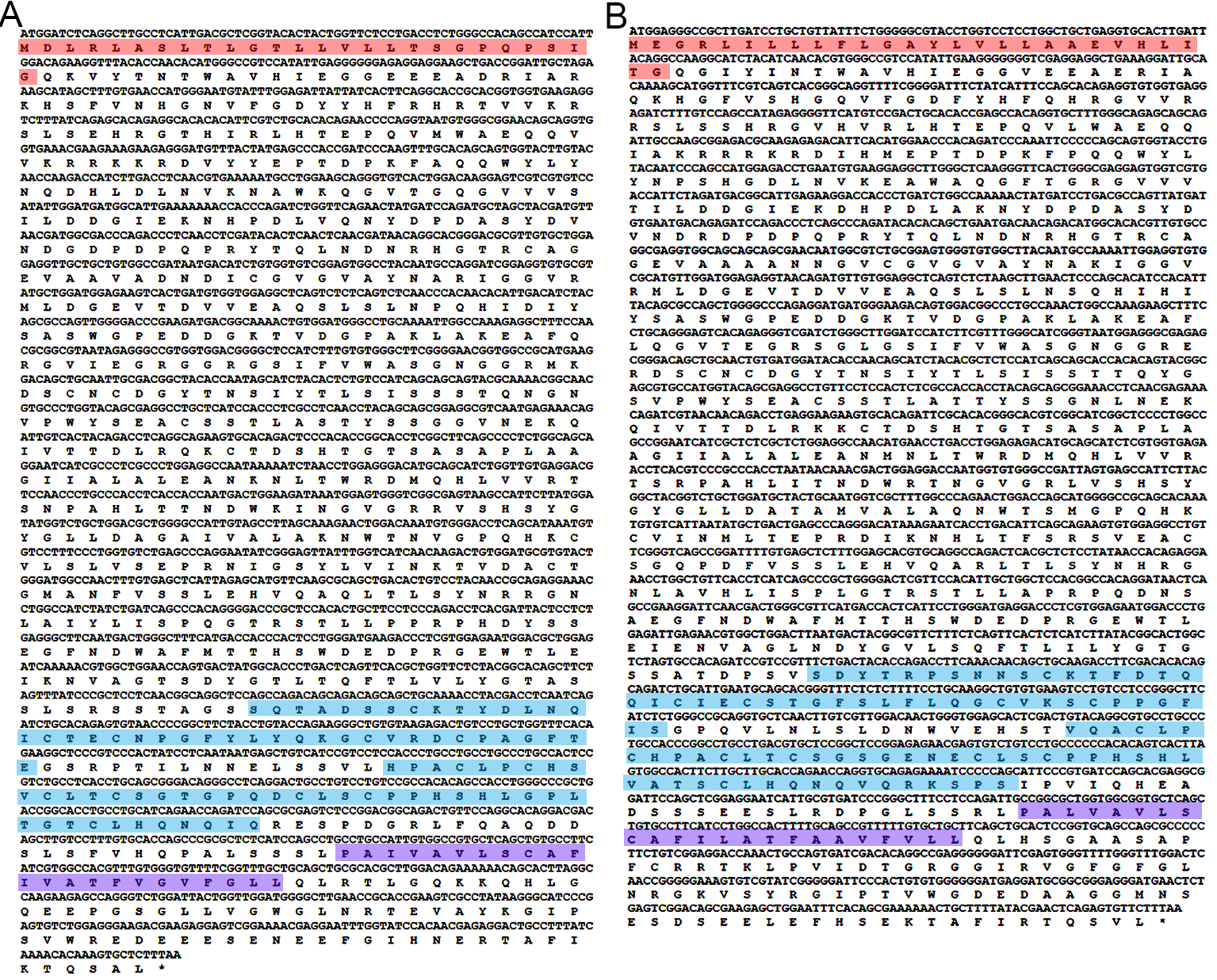

Supplement: S2 Fig — The signal peptides and transmembrane domains were bottomed with red and purple, respectively. The FU domains with furin-like cysteine rich regions were bottomed with blue. (TIF) [file ppat.1011222.s003.tif]

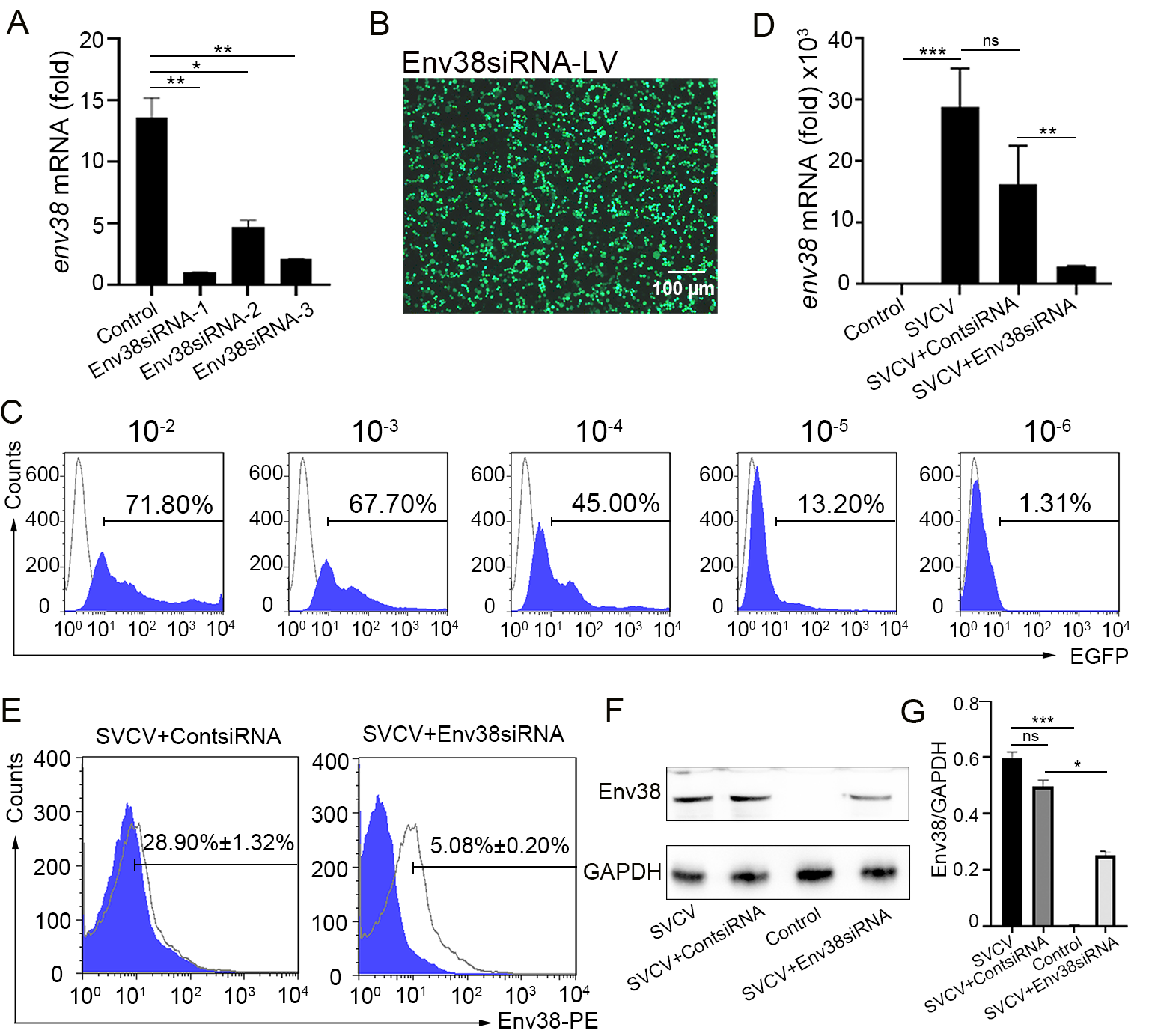

Supplement: S3 Fig — (A) RT-qPCR analysis for the efficacy of siRNAs targeting env38 mRNA in HEK293T cells. pSUPER construct containing the scrambled siRNA was used as the negative control. (B and C) Examination on the titers of the constructed Env38siRNA-LV in HEK293T cells based on EGFP fluorescence observed under a fluorescence microscope (B) and analyzed by FCM analysis (C). Fluorescence images were captured using Zeiss Axiovert 40 CFL with 100 × original magnification. (D-F) Examination on the efficiency of Env38siRNA-LV-mediated knockdown of Env38 in leukocytes from spleen, head kidney and peripheral blood via RT-qPCR (D), FCM analysis (E) and in spleen tissue via Western blot (F) under SVCV (105 TCID50) stimulation. (G) Grayscale quantization of Env38 protein examined by Western blot, in which the normalization was complied by the gray value ratio of the target Env38 protein and the internal reference GAPDH protein. Nonrelated control groups were administered with scrambled siRNA-LV. Negative control groups received mock PBS. RT-qPCRs were run in combination with the endogenous β-actin control. Error bars represented SEM. (*p < 0.05; **p < 0.01; ***p < 0.001; ns, no significant difference). (TIF) [file ppat.1011222.s004.tif]

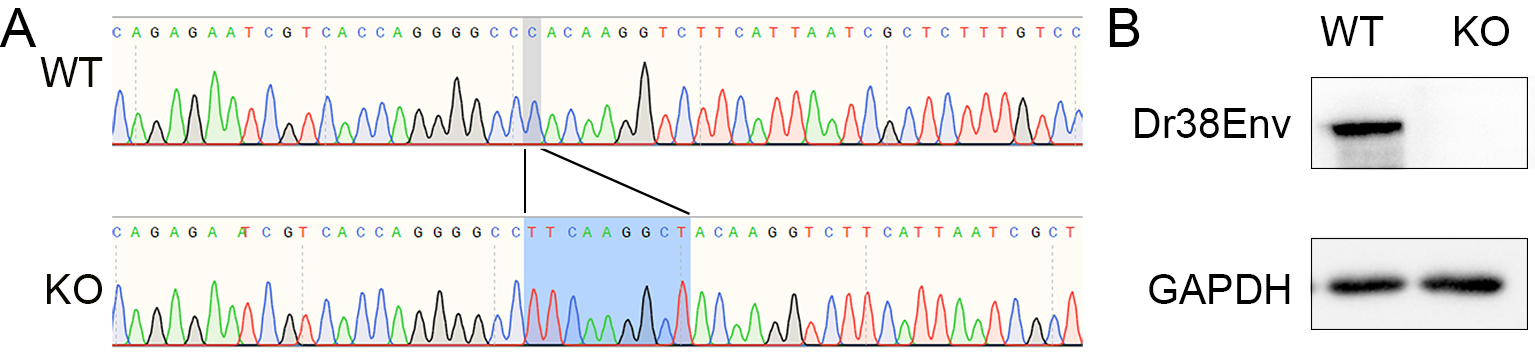

Supplement: S4 Fig — (A) Sequencing chromatograms of env38 nucleicacid sequence in knockout zebrafish. The shadows presented the alternative bases between wild type (WT) and knockout (KO) zebrafish. The targeted “C” was replaced by “TTCAAGGCT”. (B) Western blot analysis of Env38 protein in spleen tissues of WT and KO zebrafish. (TIF) [file ppat.1011222.s005.tif]

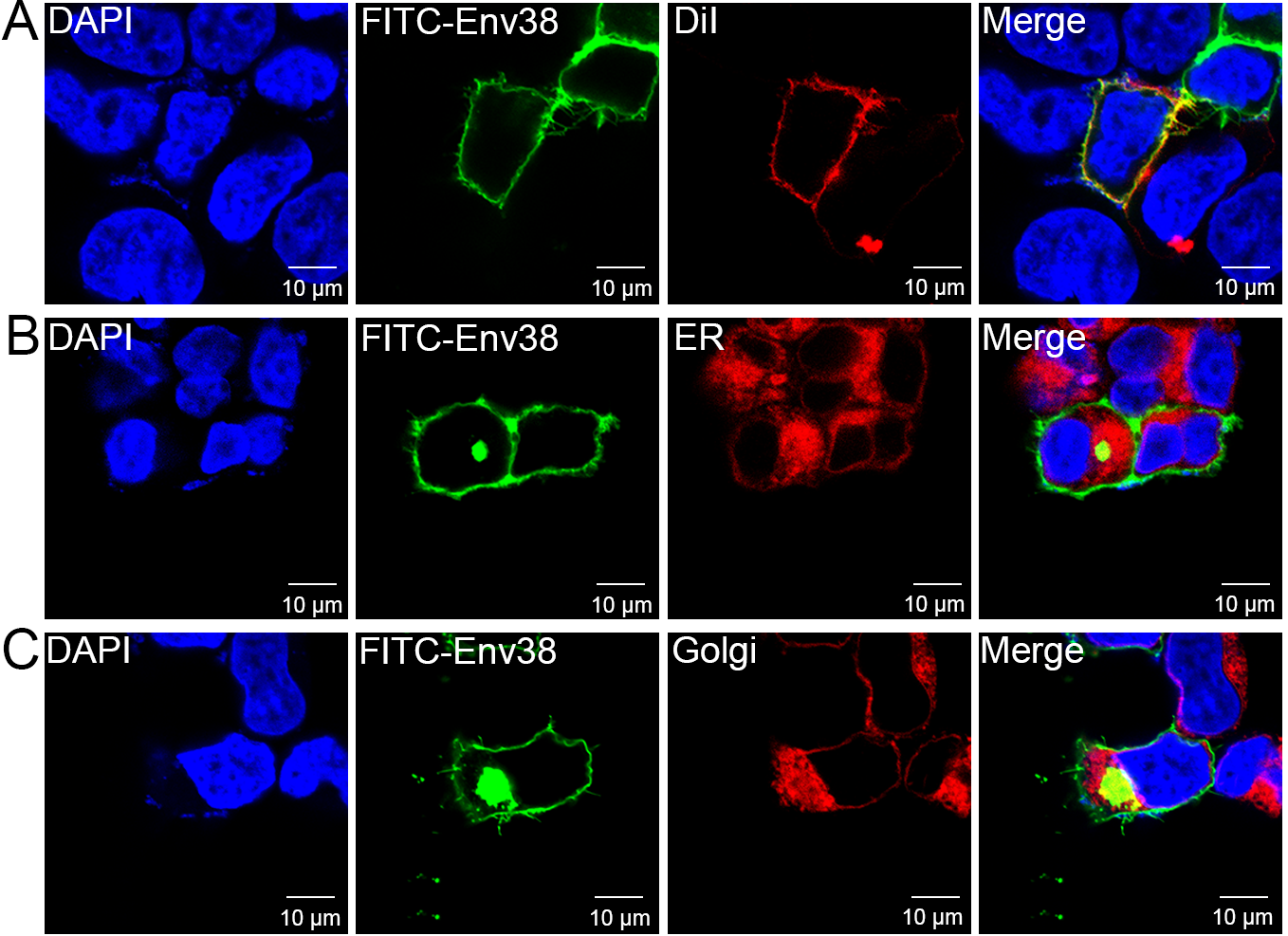

Supplement: S5 Fig — HEK293T cells were transfected with the recombinant expression plasmid of pcDNA3.1-Flag-Env38-LTR (0.6 μg/ml) for 48 h, and then fixed and labeled with mouse anti-Env38 Ab (1:500), followed by FITC-conjugated goat anti-mouse IgG (1:1,000). Next, the cells were stained with the cell membrane probe DiI (A), ER-tracker (B) or Golgi-tracker (C) and nuclei probe DAPI. The blue, green, and red fluorescence images showed DAPI-labeled nuclei, FITC-labeled Env38 protein, and DiI-labeled cell membrane, ER-tracker-labeled ER, and Golgi-tracker-labeled Golgi apparatus. Fluorescence images were captured using a Laser scanning confocal microscope (FV3000) with 60 × oil glass. (TIF) [file ppat.1011222.s006.tif]

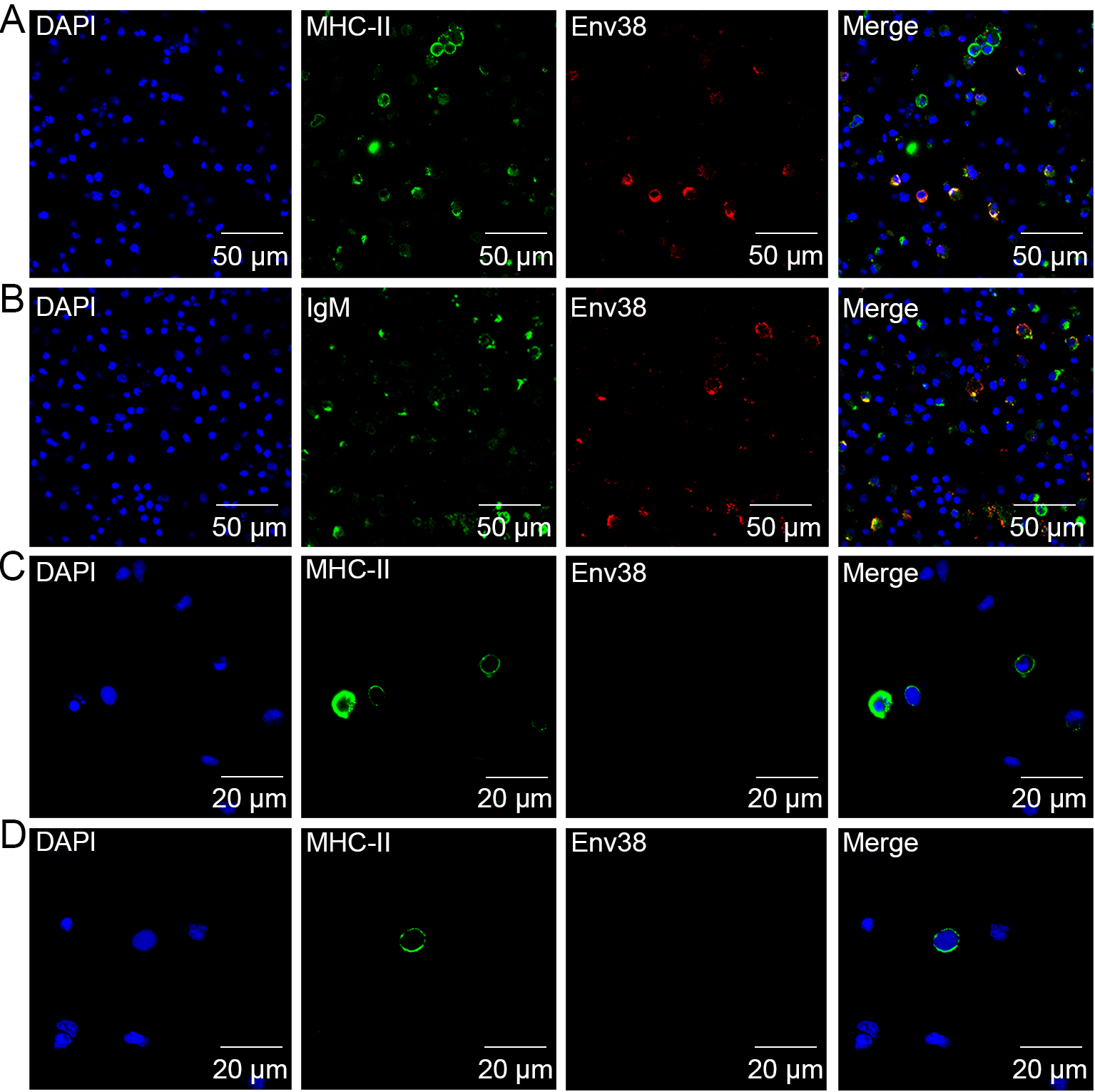

Supplement: S6 Fig — (A and B) Immunofluorescence staining for the distribution of Env38 on MHC-II+ and IgM+ cells in leukocytes from spleen, head kidney and peripheral blood of zebrafish with SVCV (105 TCID50) stimulation. Cells were stained with mouse anti-Env38 Ab (1:500) together with rabbit anti-MHC-IIα Ab (1:500) (A) or mouse anti-Env38 Ab (1:500) together with rabbit anti-IgM Ab (1:500) (B), followed by Alexa Fluor 594-conjugated goat anti-mouse IgG (1:1,000) and FITC-conjugated goat anti-rabbit IgG (1:1,000), respectively. (C and D) Immunofluorescence staining for the absence of Env38 protein in leukocytes of zebrafish without SVCV stimulation. The leukocytes were sorted from spleen, head kidney and peripheral blood and fixed and then permeabilized with Saponin (C) or not (D). Next, the leukocytes were labled with mouse anti-Env38 Ab (1:500) and rabbit anti-MHC-IIα Ab (1:500), followed by Alexa Fluor 594-conjugated goat anti-mouse IgG (1:1,000) and FITC-conjugated goat anti-rabbit IgG (1:1,000). DAPI stain showed the location of the nuclei. Fluorescence images were captured using a Laser scanning confocal microscope (FV3000) with 60 × oil glass. (TIF) [file ppat.1011222.s007.tif]

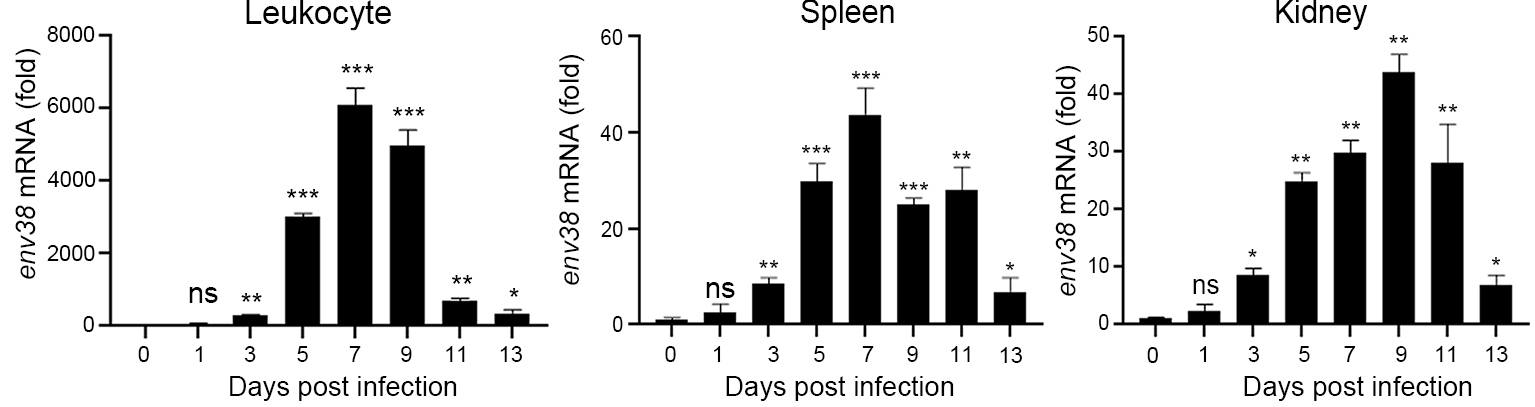

Supplement: S7 Fig — The kinetic expression patterns of the env38 in spleen, head kidney tissues and leukocytes from zebrafish under stimulation with SVCV (105 TCID50) were examined by RT-qPCR. Each sample was obtained from at least 10 fish. RT-qPCRs were run in combination with the endogenous β-actin control. Error bars represented SEM. (*p < 0.05; **p < 0.01; ***p < 0.001; ns, no significant difference). (TIF) [file ppat.1011222.s008.tif]

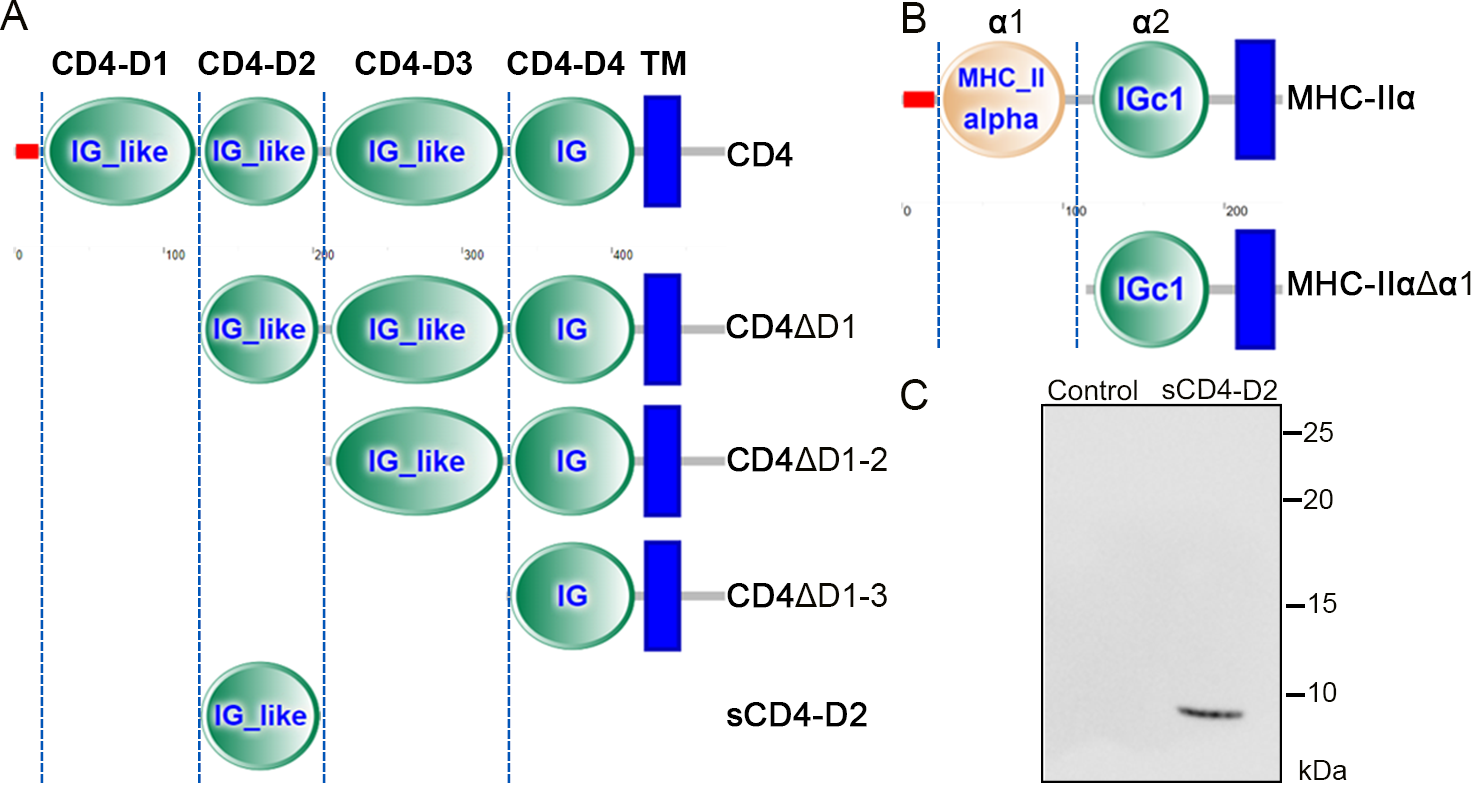

Supplement: S8 Fig — (A) Schematic diagram of the wild type CD4, truncated proteins with deletions of IgG domains of CD4 and the truncated extracellular CD4-D2 domain protein (sCD4-D2). (B) Schematic diagram of the wild type and truncated structures of MHC-IIα proteins. The architecture analysis of CD4 and MHCIIα proteins were conducted by SMART program. (C) Western blot analysis of the recombinant sCD4-CD2 protein with anti-Flag Ab (1:5,000) from supernatant of HEK293T cells transfected with the pcDNA3.1-His-Flag-CD4-D2 recombinant constructs or an empty control construct. (TIF) [file ppat.1011222.s009.tif]
