# Supplementary material for: Essential role of an ERV-derived Env38 protein in adaptive humoral immunity against an exogenous SVCV infection in a zebrafish model
Source: PLoS Pathog. 2023 Apr 4;19(4):e1011222. doi: 10.1371/journal.ppat.1011222 (PMC10072453; doi:10.1371/journal.ppat.1011222)
Supplement: Attachment — Submitted filename: point-by-point response.pdf [file ppat.1011222.s011.pdf]

Point-by-point Responses to reviewers' comments

We gratefully appreciate the reviewers and editors for their time and insightful comments, which have enabled us to improve the quality of our work. Each suggested revision and comment have been accurately incorporated and considered. Our responses and revisions are indicated beneath the comments point by point.

Part I - Summary

Reviewer #1:

The manuscript demonstrated the function of Env38 in zebrafish adaptive immunity against SVCV infection. Env38 is a glycosylated membrane protein mainly distributed on MHC-II+ APCs, which could be induced by zebrafish IFNφ1. Through a series of molecular and biochemical analyses from in vivo and in vitro experiments, the authors found that Env38 interacted with both MHC-IIα of APCs and CD4 of CD4+ T cells to form MHC-TCR-CD4 complexes, which activated CD4+ T cells, and promoted the proliferation of IgM+/IgZ+ B cells. The authors are the first to discover the role of Env protein in adaptive humoral immunity, and the results are well presented.

Reviewer #2:

Accumulating evidence suggests that endogenous retroviral elements have been co-opted by host cells. Here, Hong and colleagues describe a fascinating example of how a retroviral envelope protein (termed Env38) mediates adaptive immune responses against Spring viraemia of carp virus (SVCV) in zebra fish. The authors

convincingly show that Env38 expression is induced in response to SVCV infection and IFNφ1 stimulation. Using an elegant combination of blocking, knock-down and knockout experiments, the authors show that Env38 is required for efficient SVCV-induced proliferation of CD4 + T cells and IgM + /IgZ + B cells, as well as IgM/IgZ antibody production. Notably, the authors also demonstrate that Env38 cross-links MHC-II and CD4, and they identify the protein domains involved in this interaction. One major strength of the study is the use of several alternative approaches (e.g. antibody-mediated blocking, knockdown and knockout experiments) and the validation of in vitro findings in zebra fish models in vivo. The manuscript contains an impressive amount of data. While I am listing quite a number of points and detailed comments below, most of my concerns can be addressed by textual changes.

Reviewer #3:

In this study, the authors investigate the role of an envelope protein (Env38) derived from an endogenous retrovirus (ERV) in zebrafish adaptive immunity against Spring viraemiaof carp virus (SVCV). They observe that Env38 is a glycosylated membrane protein mainly distributed on MHC-II+ antigen-presenting cells. Antibody blockade and Env38 knockdown and knockout assays show that disruption of Env38 expression or function impairs the activation of SVCV-induced CD4+ T cells and zebrafish defense against SVCV challenge. The authors present data showing that Env38 activates CD4+ T cells by promoting the formation of a pMHC-TCR-CD4 complex via cross-linking MHC-II and CD4 molecules between the APCs and CD4+ T cells.

The expression of Env38 appears to be strongly induced by zebrafish interferons. The study implicates Env38 in host immune defense against an exogenous virus.

Overall, the study represents a large amount of high-quality work that provides new insights into the role of endogenous retroelements in adaptive immunity. In my view, the study will be of interest to a wide audience. I have mostly minor comments for improvement of the manuscript.

Part II – Major Issues: Key Experiments Required for Acceptance

Reviewer #1:

The manuscript is very informative, which is a collection of too vast an array of different findings. Please re-write it with a clear story. Such as these results involved in the glycosylation and cleavage-processing of Env38 distracted my attention. In fact, the authors did not provide these data that the glycosylation and cleavage-processing of Env38 was involved in the function of Env38 in host adaptive immunity.

Response: In accordance with reviewer's helpful suggestions, we have reorganized the manuscript to improve the writing. Considering that the results involved in the glycosylation and cleavage-processing of Env38 protein are actually an integral of the molecular characterization of Env38, these results were reorganized into the first paragraph of "Molecular characterization of ERV-E5.1.38-DanRer-Env (Env38)". In addition, the results involved in the "Generation of Env38 siRNA-encoding LV and knockout zebrafish" were reorganized into the second paragraph of "Preparation of recombinant proteins and polyclonal antibody" considering their relevance in content.

Similarly, the results involved in the "Subcellular localization of Env38 protein" were combined with the results of "Tissue and cellular distribution of Env38".

Reviewer #2:

1. Fig. 3: For the localization analyses shown in this figure, Env38 was cloned into pEGFPC1, resulting in an EGFP-Env38 fusion protein. It is highly likely that fusion of EGFP to the N-terminus of Env38 affects the normal subcellular localization of the latter. Furthermore, the N-terminal EGFP may also be cleaved off if it was indeed fused to the N-terminal signal peptide of Env. The authors should use untagged or C-terminally tagged (His-tag, FLAG-tag, etc.) Env38 instead of EGFP-Env38 chimeras to determine the subcellular localization of Env38 in the presence and absence of SVCV.

Response: We appreciate reviewer's insightful comments. As per reviewer's helpful suggestions, we have used non-EGFP tagged Env38 to determine its subcellular localization with Env38-specific antibody. For this procedure, HEK293T cells were transfected with the recombinant pcDNA3.1-Flag-Env38-LTR plasmid and then fixed, blocked and labeled with mouse anti-Env38 Ab followed by FITC-conjugated goat anti-mouse IgG, and stained with DiI, or ER-tracker Red or Golgi-tracker Red, then the cells were stained with DAPI. As expected, the Env38 signal was substantially located on the plasma membrane, as well as on/in ER and Golgi apparatus. Meantime, we have also used the C-terminally tagged Env38-EGFP instead of the N-terminally tagged Env38 in HEK293T and EPC cells as suggested. The results also showed significant localization of Env38 on the plasma membrane, and ER and Golgi

apparatus. These observations are consistent with that observed in the examination by using non-EGFP tagged Env38 protein. In addition, Env38 was more significantly distributed on the plasma membrane of EPC cells under stimulation with SVCV. Accordingly, we have added these improved data in our revised manuscript (Fig 3 and S5 Fig) for better presentation. Here, we would like to present these results as follows.

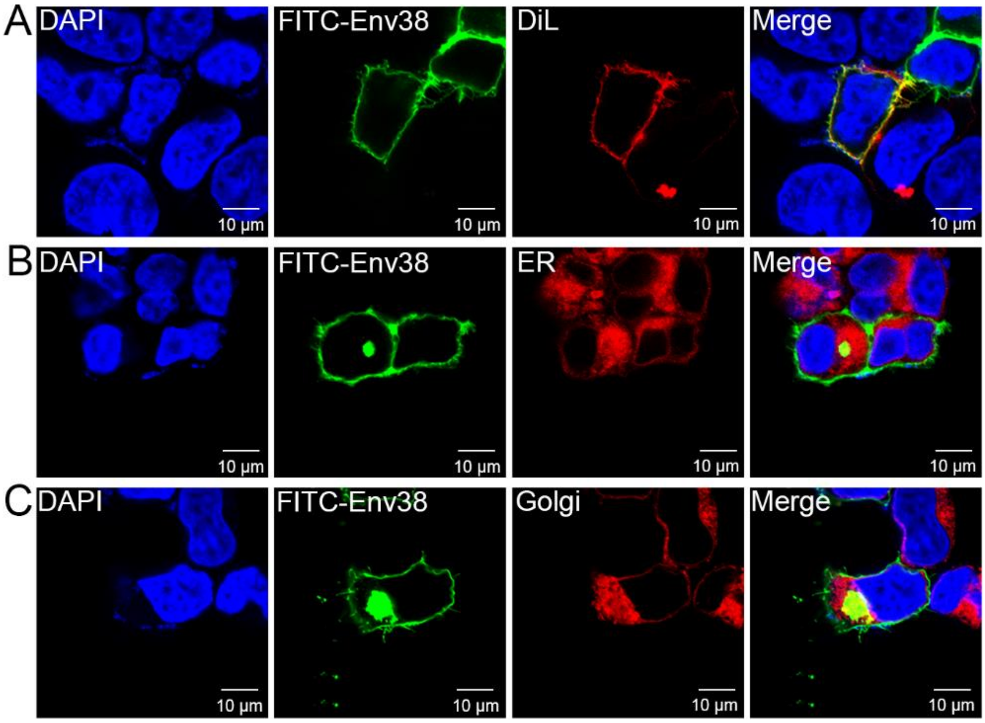

Subcellular localization analysis of non-EGFP tagged Env38 in HEK293T cells. (A-C) HEK293T cells were transfected with the recombinant expression plasmid of pcDNA3.1-Flag-Env38-LTR (0.6 μg/ml) for 48 h, and then fixed by 4% PFA, blocked with 2% BSA, labeled with mouse anti-Env38 Ab (1:1:500) followed by FITC-conjugated goat anti-mouse IgG (1:1,000), and stained with cell membrane probe DiI (1:200) (A), or ER-Tracker Red (1: 2,000) (B), or Golgi-Tracker Red (1:200) (C). Next, the cells were stained by DAPI (100 ng/ml). The blue, green, and red fluorescence images showed DAPI-labeled nuclei, FITC-labeled Env38 protein, and

DiI-labeled cell membrane, ER-tracker-labeled ER, or Golgi-tracker-labeled Golgi apparatus. Fluorescence images were captured using Laser scanning confocal microscope (FV3000) with 60 × oil glass.

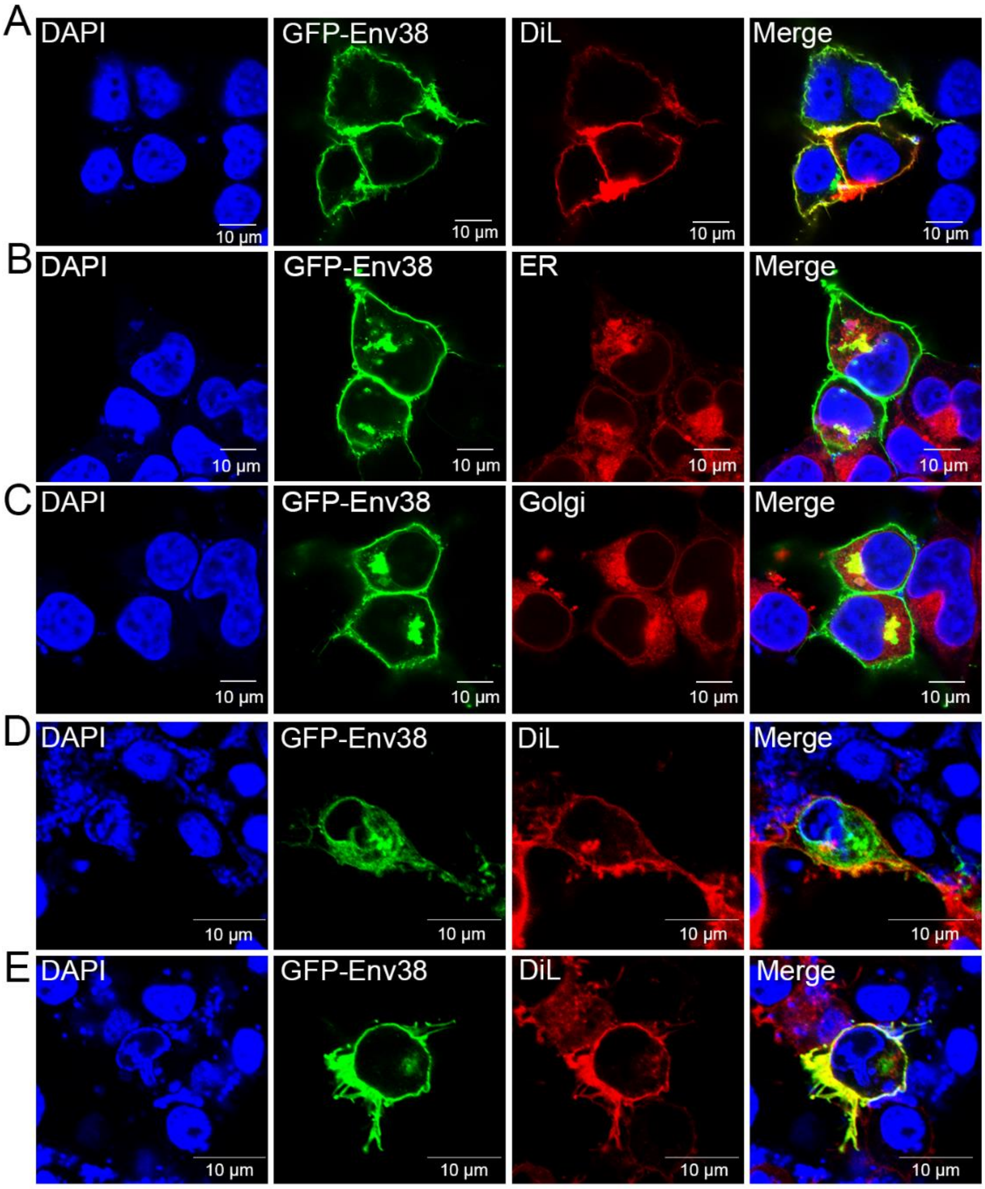

Subcellular localization analysis of C-terminally EGFP-tagged Env38 in HEK293T and EPC cells. (A-C) HEK293T cells were transfected with the recombinant expression plasmid of pEGFPN1-Env38 (0.6 μg/ml) for 48 h, and then fixed by 4% PFA and stained with cell membrane probe DiI (1:200; Beyotime) (A), or

ER-Tracker Red (1: 2,000) (B), or Golgi-Tracker Red (1:200) (C). Next, the cells were stained by DAPI (100 ng/ml). (D and E) EPC cells were transfected with the recombinant expression plasmid of pEGFPN1-Env38 (0.6 μg/ml) for 24 h and stimulated with SVCV (100 $TCID_{50}$) (E) or not (D) for another 24 h, and then fixed by 4% PFA and stained with cell membrane probe DiI (1:200) and nuclei probe DAPI (100 ng/ml). The blue, green, and red fluorescence showed DAPI-labeled nuclei, Env38–GFP fusion protein, and DiI–labeled cell membrane. Fluorescence images were captured using Laser scanning confocal microscope (FV3000) with 60 × oil glass.

Fig. 4C/D: Can the authors include a control without SVCV stimulation to validate the relocalization of Env38 they propose? A comparison of permeabilized and unpermeabilized cells may also be helpful in this case.

Response: In accordance with the reviewer's insightful suggestions, we have included a control group with permeabilized and unpermeabilized leukocytes without SVCV stimulation. Actually, we found that Env38 was strictly induced by SVCV stimulation. It was hardly expressed in the leukocytes of zebrafish without SVCV infection as examined by RT-qPCR and Western blot analysis at mRNA and protein levels (shown in S3D Fig and Fig 5C). Consistent with these observations, immunofluorescence staining analysis also showed that Env38 was hardly colocalized with MHC-II on/in unpermeabilized and permeabilized control leukocytes without SVCV stimulation. However, substantial colocalization of Env38 and MHC-II was observed on/in the leukocytes of zebrafish upon SVCV infection (shown in Fig 4 and S6A, S6B Fig). It

suggested the induced expression and membrane-localization of Env38 in the MHC-II$^+$ APCs during their activation/antigen-presentation in response to SVCV stimulation. We have added the control data in the revised manuscript (S6C and S6D Fig). Here, we would like to present these results as follows.

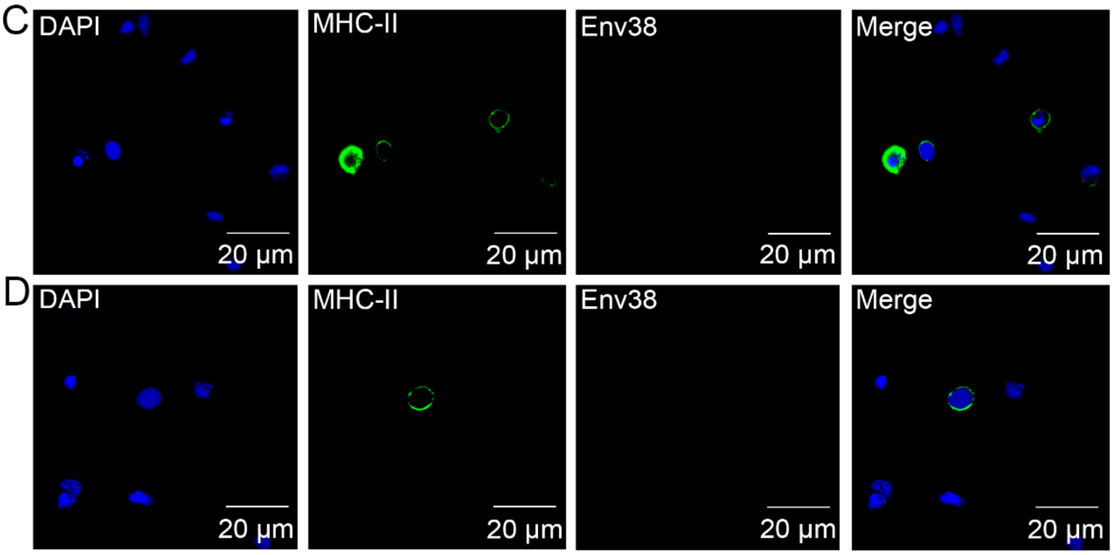

Immunofluorescence staining for the absence of Env38 protein in leukocytes of zebrafish without SVCV stimulation. The leukocytes were sorted from spleen, head kidney and peripheral blood and then fixed and permeabilized with Saponin (0.1%) (C) or not (D). Next, the leukocytes were labled with mouse anti-Env38 Ab (1:500) and rabbit anti-MHC-IIα Ab (1:500), followed by Alexa Fluor 594-conjugated goat anti-mouse IgG (1:1,000) and FITC-conjugated goat anti-rabbit IgG (1:1,000). DAPI stain showed the location of the nuclei. Fluorescence images were captured using a Laser scanning confocal microscope (FV3000) with 60 × oil glass.

2. Lines 274/275: The authors state that “Env38 might act as a trafficking transmembrane protein that underwent post translational glycosylation […] after SVCV stimulation”. Western blotting needs to be performed to validate the hypothesis

that SVCV stimulation alters the glycosylation of Env38.

Response: We regret for this inappropriate description. Here, we suggest that SVCV stimulation might promote the translocation of Env38 from Golgi apparatus onto plasma membrane, but do not alter the glycosylation of Env38. We have improved this sentence in the revised manuscript to avoid misleading (lines263-265).

3. Fig. 8A: Can the authors include a survival curve of unimmunized KO animals?

Response: We appreciate the reviewer's advice. We have added the survival curve of unimmunized KO zebrafish in our revised manuscript in Fig. 9A (Fig 8A in original manuscript, and Fig 9A in revised manuscript).

Reviewer #3: (No Response)

Part III – Minor Issues: Editorial and Data Presentation Modifications

Reviewer #1:

1. In Fig 2E, Furin enzymes could cleave Env38 into SU and TM subunits, why WB can only detect TM subunit but not SU subunit? In Fig 2F, how to explain the undetectable expression of furina and furinb in Env38+ cells?

Response: Thanks for the reviewer's insightful comments. We used the recombinant pcDNA3.1-Flag-Env38-LTR expression plasmid in this experiment, the Flag-tag is in the C-terminus of Env38. In WB analysis, when incubated with anti-Flag Ab, only the cleaved TM subunit that nears the C-terminus and the intact Env38 protein with both SU and TM subunits can be detected. As per reviewer's comment, we have added a WB analysis to show both of the cleaved SU and TM subunits of Env38 by using

anti-Env38 antibody. We have supplemented these data in the revised manuscript in Fig 2D. Here, we would like to present these data as follows. In Fig 2G (Fig 2F in original manuscript, and Fig 2G in revised manuscript), we did not detect the expression of FurinA and FurinB in Env38$^+$ cells. This observation indicated that the *furina* and *furinb* genes are silenced in Env38$^+$ cells. However, further study is required to understand the mechanisms underlying *furina* and *furinb* gene silence in Env38$^+$ cells, such as the epigenetic regulatory mechanism, considering its close association with the silence and activation of genes. We suppose that the repression of FurinA and FurinB in Env38$^+$ cells may be of biological significance for Env38. In this case, repression of FurinA/B expression can prevent furin proteases from cleaving Env38 at the existing furin cleavage site. This behaviour can avoid the release of fusion peptide and exposure of immunosuppressive domain (ISD) from the Env38 protein, which benefits the stimulatory role of Env38 in T cell activation by help in establishing the immune synapse complex between Env38$^+$ APCs and CD4$^+$ T cells, but not in the formation of syncytial cells by promoting the membrane-membrane fusion between Env38$^+$ APCs and CD4$^+$ T cells.

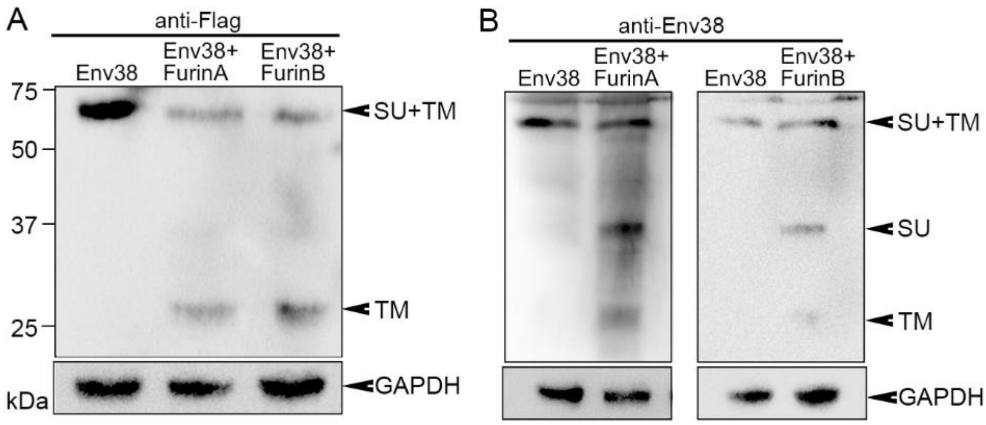

Western blot analysis for the detection of enzymatic cleavage of Env38 protein by

Furins. Env38 with a Flag-tag at the C-terminus and FurinA or FurinB were coexpressed in HEK293T cells. Two protein bands corresponding to the intact Env38 protein and the TM subunit with molecular weights of approximately 60 kDa and 25 kDa were detected with anti-Flag Ab (A) and three protein bands corresponding to the intact Env38 protein, the SU subunit and the TM subunit with molecular weights of approximately 60 kDa, 35 kDa and 25 kDa were detected with mouse anti-Env38 Ab (B). GAPDH was used as a loading control.

2. Lines 301 and 1162: How did the authors conclude that the leukocytes they isolated were lymphatic and myeloid leukocytes?

Response: Thanks for pointing out this issue. In FCM analysis (such as Fig 4E in original manuscript, and Fig 5C in revised manuscript), the leukocytes were analyzed by the size (forward scatter; FSC) and granularity (side scatter; SSC) characteristics, and the lymphatic and myeloid leukocytes were gated according to the strategy that has been well established in zebrafish as described previously (Trede et al., Immunity. 2004, 20(4):367-79; Traver et al., Nat Immunol. 2003, 4(12):1238-46).

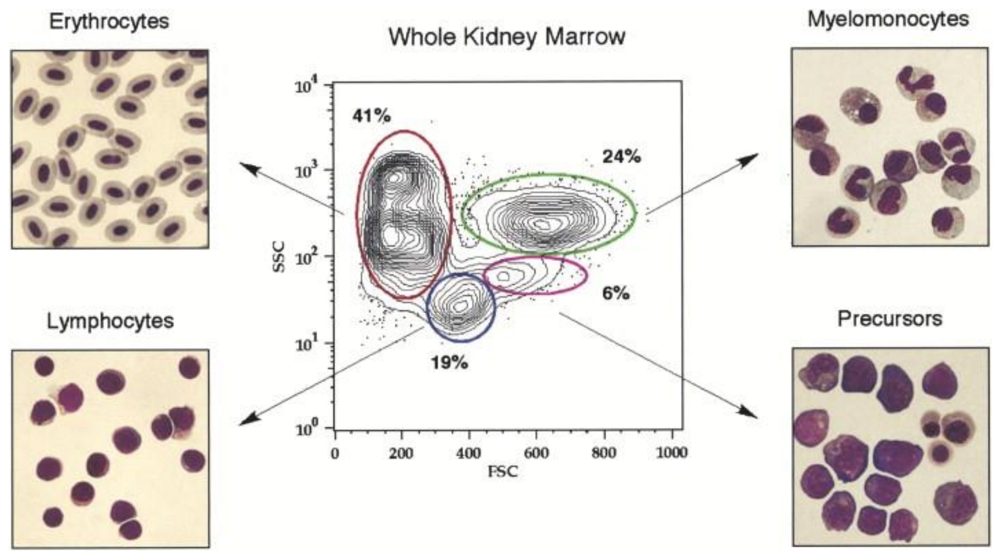

Suspensions of adult zebrafish kidney marrow cells form distinct populations when analyzed by size (forward scatter; FSC) and granularity (side scatter; SSC). Sorting of each population reveals that cells within the red gate are comprised of only mature erythrocytes (upper left panel), the blue gate contains only lymphocytes (lower left panel), the purple gate contains immature precursors of all mature blood lineages (lower right panel), and that the green gate contains only myelomonocytic cells (upper right panel). Percentages indicated in each population may vary between different strains of fish and different aquatic habitats (cited from Traver et al., Nat Immunol. 2003, 4(12):1238-46).

References

Trede NS, Langenau DM, Traver D, Look AT, Zon LI. The use of zebrafish to understand immunity. Immunity. 2004;20(4):367-79. doi: 10.1016/s1074-7613(04) 00084-6. PubMed PMID: 15084267.

Traver D, Paw BH, Poss KD, Penberthy WT, Lin S, Zon LI. Transplantation and in vivo imaging of multilineage engraftment in zebrafish bloodless mutants. Nat Immunol. 2003;4(12):1238-46. doi: 10.1038/ni1007. PubMed PMID: 14608381.

3. In Fig 5B, the IgM produced in SVCV+contsiRNA group was higher than that of SVCV group, and the mRNA level of Env38 in SVCV group was higher than SVCV+contsiRNA group. How to interpret these results?

Response: We thank the reviewer for pointing out these issues. In siRNA-mediated knockdown assay, the IgM produced in SVCV+contsiRNA group showed higher than that of SVCV group, however, the IgM produced in SVCV+Env38siRNA group was

markedly declined compared with that of SVCV group. We suppose that zebrafish in SVCV+contsiRNA group may receive an additional stimulation from contsiRNA. In this case, the contsiRNA may act as an external stimulus to activate innate immune signaling pathways, that may have an additional effect on the activation of adaptive immunity to a certain extent. In addition, statistical analysis showed that there is no significant difference (p= 0.226033) in mRNA/protein level of Env38 between SVCV group and SVCV+contsiRNA group, despite it looks a little high in SVCV group compared with that of SVCV+contsiRNA group in S3D Fig (S2D Fig in original manuscript, and S3D Fig in revised manuscript).

4. In Fig 8A: To confirm that these zebrafish died from SVCV infection, the authors should also investigate the viral loads in the wildtype and knockout zebrafish.

Response: As per reviewer's suggestion, we have added viral load examinations in the wildtype and knockout zebrafish in the revised manuscript in Fig. 9B (Fig 8 in original manuscript, and Fig 9 in revised manuscript).

5. In Fig 9G, the Env38 protein band expressed in E.coli (48 kDa) should be smaller than that expressed in sf9 cells (60 kDa).

Response: We regret for this clerical error, and we have revised the annotation in the figure panels in our revised manuscript in Fig. 10G (Fig 9G in original manuscript, and Fig 10G in revised manuscript).

6. Line 335: Please define CD4+ Tsvcv when it first appears.

Response: We have defined $CD4^+$ Tsvcv in the revised manuscript (lines 326-328) as suggested. $CD4^+$ Tsvcv was defined as the antigen-specific $CD4^+$ T cells ($CD4^+$ Tsvcv)

sorted by FCM from the leukocytes of zebrafish vaccinated by injection with inactivated SVCV (10^6 TCID50).

Reviewer #2:

1. Lines 257-259: The authors state that, minimal cleavage of the Env38 protein was detected in the spleen, head kidney, and leucocytes of zebrafish stimulated with SVCV (10^5 TCID50) (Fig 2B).” However, no such band is visible in Fig. 2B. Can the authors show the same blot in a higher intensity to make the cleaved 25 kDa form visible?

Response: We regret for this inappropriate description. Here, we want to express the idea of “negative reactions”. We have replaced “minimal” with “no” in the revised manuscript (line 210).

2. Fig. 2: In many cases, individual lanes were stitched together and it remains unclear whether they originate from the same blot. In Fig. 2C, for example, it remains unclear whether untreated and tunicamycin treated HEK293T cell samples were run on the same blot. Furthermore, the PNGase F treated Sf9 lysates should be shown next to an untreated Sf9 negative control. Instead, they are shown next to an untreated HEK293T cell sample, which may be misleading. Similarly, it remains unclear whether the samples in Fig. 2E were run on the same blot. In this case, it would have been nice to validate expression of Furin in lanes 2 and 3 instead of separate samples. Finally, detection of a housekeeping protein (e.g. GAPDH or beta-actin) may enable a better comparison of the samples in Figs. 2C and 2E.

Response: We appreciate the reviewer’s kind advice. As per reviewer’s suggestions,

we have re-prepared and run the untreated and tunicamycin treated HEK293T cell samples on the same blot and detected with anti-flag in WB analysis, and detection of GAPDH was supplemented as internal references. We have also re-prepared and run the undigested and PNGase F digested Env38 protein samples derived from the same batch of purified Sf9-expressed protein on the same blot and detected with anti-His in WB analysis. In addition, we have re-prepared and run the Env38 protein samples co-expressed with FurinA or FurinB or not in HEK293T cells on the same blot; and detection of GAPDH was added as internal references. We have replaced the original figures with these new images in the revised manuscript in Fig. 2B and Fig. 2D. Here, we would like to present these improved data as follows.

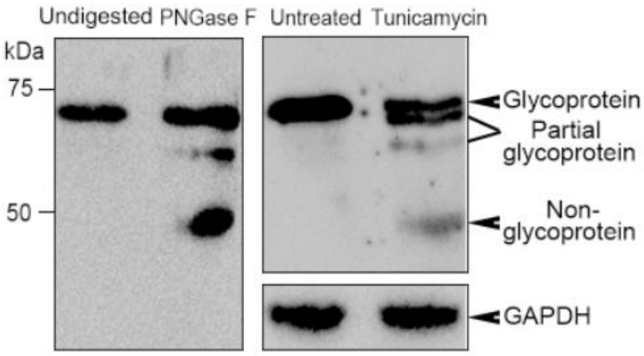

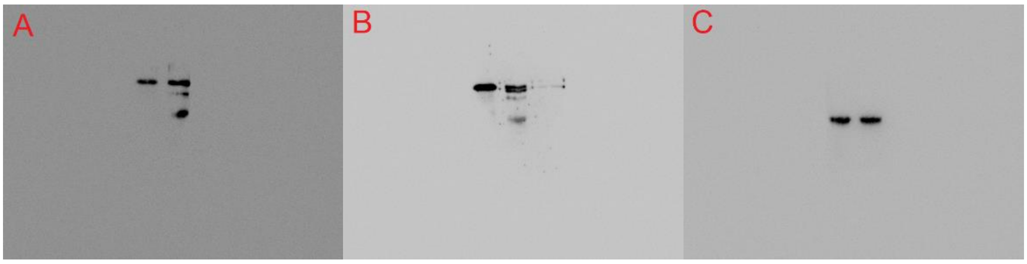

Western blot analysis for N-glycosylation of Env38 protein without (control) and with treatment of PNGase F or tunicamycin in Env38-expressing Sf9 cells or HEK293T cells. Fig A is the original image of undigested and PNGase F treated Env38; Fig B is the original image of untreated and Tunicamycin treated Env38; Fig C is the original

image of GAPDH.

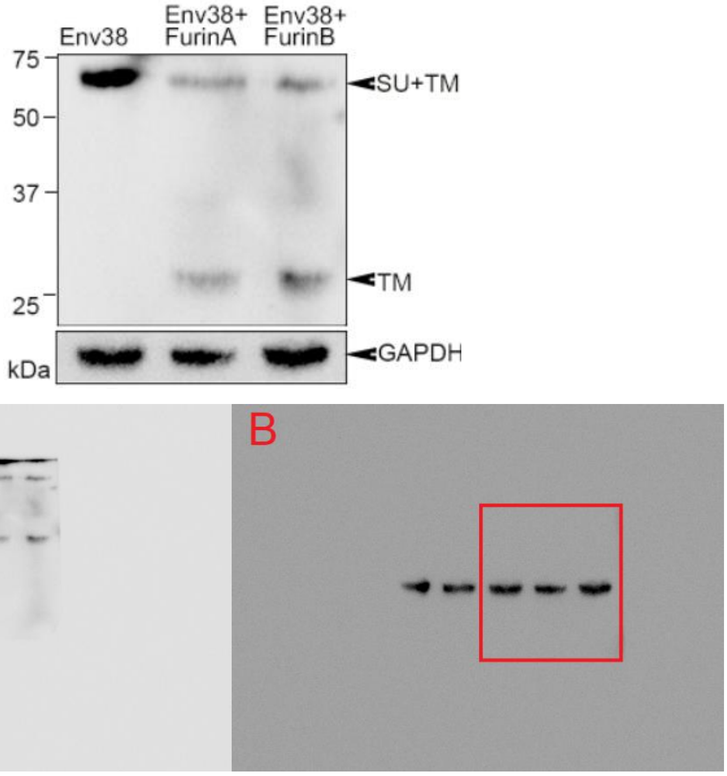

Western blot analysis for the expression and FurinA/FurinB cleavage of Env38 protein. Coexpression of Env38 with FurinA/FurinB in HEK293T cells resulted in cleavage of Env38 into TM subunit with an expected molecular weight of 25 kDa as detected with anti-flag Ab. Fig A is the original image of Env38 protein before and after FurinA/FurinB treatment. Fig B is the original image of GAPDH.

3. Line 46: It has been proposed that endogenous retroviruses may also be found in plants (e.g., Laten and Gaston, Plant Transposable Elements, Topics in Current Genetics 24, DOI 10.1007/978-3-642-31842-9_6, 2012). Thus, statements regarding the exclusive presence of ERVs in vertebrates may be toned down accordingly.

Response: We appreciate the reviewer’s comments. We have revised the description and supplemented the reference in our revised manuscript (lines 45-46).

4. Lines 206-209: The description of zebrafish IFNφ and sCD4ΔD2 may be

mentioned at a later time point, when describing the experiments shown in Fig. 10 and S7.

Response: We have revised the description of zebrafish IFNφ and sCD4ΔD2 at a later time point in our revised manuscript (lines 476-478 and lines 459-461).

5. Line 207: The authors are referring to “CD4-D2”, rather than “CD4ΔD2”, I assume.

Response: We have revised this description in the revised manuscript (line 459).

6. Fig. S2B: A negative control may be included (optional).

Response: Thanks for the suggestion. In S3B Fig (S2B Fig in original manuscript, and S3B Fig in revised manuscript), the cells without infection of LVs were used as negative control, and no any fluorescence was detected in these control cells. Considering the limited space of S3 Fig, we wonder if the negative image could be no longer included in S3 Fig.

7. Line 242: “inhibition of glycosylation” may be more appropriate than “deglycosylation” in the case of tunicamycin.

Response: We appreciate the reviewer’s suggestion. We have revised the description accordingly (line 202).

8. Line 260, Fig. 2F: What kind of Env38+ cells are shown here?

Response: In Fig 2F (Fig 2F in original manuscript, and Fig 2G in revised manuscript), the $Env38^{+}$ cells were sorted from leukocytes of spleen, head kidney and peripheral blood of zebrafish under stimulation with SVCV (10^{5} $TCID_{50}$) through FCM sorting. We have supplemented this description in the revised manuscript (lines

212-214).

9. Line 265: The authors may briefly explain what EPC cells are and why they were used here.

Response: In accordance with the reviewer's advice, we have added explanation of EPC cells in the revised manuscript (lines 258-260). Zebrafish is cyprinids and EPC (Epithelioma papulosum cyprinid) is a carp-derived cell line that is highly sensitive to SVCV infection, and thereby is extensively used for the propagation, isolation and diagnostic assays for SVCV. Here, EPC line is used for detecting the subcellular localization changes of Env38 before and after SVCV infection.

10. The panels in Fig. 4 should be re-ordered according to their appearance and discussion in the text. In general, figures should be numbered according to the text.

Response: We thank for reviewer's careful reading. We have re-ordered the figures in our revised manuscript as suggested.

11. Fig. 4E, 6C, 6E, 7A, 7C, 7D, 8B, 8C, 10A, 10B, 10D: Isotype controls should be included (optional).

Response: We appreciate the reviewer's suggestion. Actually, non-related IgG is the isotype control to anti-Env38 in Env38 blockade groups, and scrambled contsiRNA-LV is the isotype control to Env38siRNA-LV in Env38 knockdown groups.

12. Fig. 9I, J: Detection of FLAG-Env in the IP samples is missing. This control is required to demonstrate similar pull-down efficiencies.

Response: We appreciate the reviewer's helpful suggestion. We have supplemented

the detections of FLAG-Env in the IP samples in the revised manuscript as suggested in Fig 10I (Fig 9I in original manuscript, and Fig 10I in revised manuscript).

13. Lines 342/343: “the percentage of CD4+ T cells in Env38-blockade groups was comparable to that of the negative control groups”. Better rephrase. The percentage in the Env38-blockade group was two-fold higher than that in the negative control.

Response: We appreciate the reviewer’s suggestion. We have improved this sentence in the revised manuscript (lines 334-337).

Reviewer #3:

1. Line 209: Figs. 10F and S7B are referred to out of order.

Response: We appreciate the reviewer for pointing out this issue. We have re-ordered the figures in the revised manuscript.

2. Fig. 2. It is surprising that zebrafish but not human furin is active in cleaving Env38, given that this protein contains what appears to be a consensus furin cleavage site. Perhaps the authors could comment.

Response: We appreciate the reviewer’s insightful comment. In Fig 2D (Fig 2E in original manuscript, and Fig 2D in revised manuscript), no obvious cleavage of Env38 was detected in HEK293T cells without additional expression with zebrafish FurinA or FurinB. This observation suggested that human Furin is not active in cleaving Env38. We suppose that a substrate/species-specific recognition mechanism towards Env38 may exist between the Furin proteases of humans and zebrafish. However, further study is required to clarify this hypothesis, which may largely depend on the deciphering of the structural basis of human and zebrafish Furin proteases.

3. Line 327, 370 and elsewhere: the authors state that Env38 plays an "essential" role in zebrafish antiviral innate immunity. "Essential" is an overstatement.

Response: As per reviewer's advice, we have replaced "essential" with other words, such as "important" in the revised manuscript (lines 50, 318, 363 and 529).

4. Fig. 9 is confusing. I think the basis for the confusion is that the left sides of the panels are mis-labeled "IP" (immunoprecipitation) instead of "IB" (immunoblot) as described in the methods. This should be corrected/clarified, and the figure legend should clearly explain what was done and how the figure is labeled.

Response: We thank for reviewer's careful reading. We have revised the labels of the panels, and improved the figure legend of Fig 10 (Fig 9 in original manuscript, and Fig 10 in revised manuscript) in the revised manuscript.

5. Throughout the paper, the English is in need of editing. Most errors are simple grammatical mistakes, but in other places the text is difficult to understand. For example, line 267 states that cells were "signed" when I think the authors mean "stained"; line 577, what is meant by 6HB "texture"? Line 577, the sentence "the fusion peptide was locally hydrophobic, implying that Env38 was fusion defective even though the 6HB post-fusion structure was still retained" doesn't make sense. Maybe the authors mean "locally hydrophilic"? Line 590 "non-cleavage reason"?

Response: As per reviewer's advice, we have tried our best to improve the writing as suggested.

6. In the Discussion section, the authors speculate on recognition of the furin cleavage site. If Env38 cleavage is disadvantageous to the host, why did Env38 not simply lose

the furin cleavage site?

Response: We appreciate the reviewer's insightful comment. In this study, we suggested that furin site of Env38 would not be cleaved by furin proteases in the $Env38^{+}$ leukocytes from spleen, head kidney and peripheral blood of zebrafish with SVCV infection. However, RT-qPCR results also showed that the expression of *env38* was detected in the liver, brain, heart, intestine, skin, and gill at varying degrees under normal conditions (Fig 4A in original manuscript, and Fig 5A in revised manuscript). It suggested that Env38 may potentially involve in other physiological activities, in which the cleavage of Env38 may be necessary.
