# Supplementary material for: Essential role of an ERV-derived Env38 protein in adaptive humoral immunity against an exogenous SVCV infection in a zebrafish model
Source: PLoS Pathog. 2023 Apr 4;19(4):e1011222. doi: 10.1371/journal.ppat.1011222 (PMC10072453; doi:10.1371/journal.ppat.1011222)
Supplement: Attachment — Submitted filename: Point-by-point response-R2.pdf [file ppat.1011222.s012.pdf]

Point by point Responses to reviewers' comments

We gratefully appreciate the editors and reviewers for their time and insightful comments, which have enabled us to improve the quality of our work. Each suggested revision and comment have been accurately incorporated and considered. Our responses and revisions are indicated beneath the comments point by point.

Part I - Summary

Please use this section to discuss strengths/weaknesses of study, novelty/significance, general execution and scholarship.

Reviewer #1: (No Response)

Reviewer #2: In the revised version of their manuscript, Hong and colleagues have addressed all of my initial comments. In addition to several textual changes, the authors have also included additional experimental data:

- Since an N-terminal EGFP tag may affect the subcellular localization of Env38 (or simply be cleaved off), the authors also monitored the localization of C-terminally tagged and untagged Env38. These new data confirm the authors' initial conclusions, and the respective images have been included in the revised version of their manuscript.

- Importantly, Hong and colleagues have also included a survival curve of unimmunized KO animals in Fig. 9A. This important control demonstrates that deletion of Env38 also significantly reduces survival in non-immunized animals. Thus, Env38 KO does not specifically attenuate protection upon immunization, and the statements in lines 397-403 need to be changed accordingly.

Response: We thank the reviewer for pointing out this issue. As advised, we have improved the statements in the revised manuscript, including in Results section (lines 403-407) and Figure legend of Fig.9A (lines 1261-1264 and lines 1266-1267) accordingly.

- The authors have also detected FLAG-Env in the IP samples in Fig. 10I to demonstrate similar pull-down efficiencies.

- Finally, the authors have re-run several of their Western blot and loaded all samples together with their corresponding controls. The original images of the Env38 cleavage blot (page 22 of the rebuttal) suggest that Env38 and GAPDH were not detected on the same membrane since 3 and 5 lanes are shown, respectively. This should be clarified. Furthermore, it still remains unclear whether Furin expression (Fig. 2E) and Env38 cleavage (Fig. 2D) were detected in the same samples/blots.

Response: Thanks for the comments. The Env38 and GAPDH were detected on the same membrane. Because of the large differences in protein expression productivity and molecular weight which would interfere with each other, we detected GAPDH and Env38 respectively. We first detected GAPDH with anti-GAPDH antibody (Invitrogen), and then stripped the anti-GAPDH antibody with Stripping Buffer (Thermo Scientific). Next, the same membrane was re-blocked, and the membrane of lanes 3-5 was cut and incubated with anti-flag antibody (Invitrogen) to detect Env38-flag; and the membrane of lanes 1-2 was used to detect Env38 and Furin A-cleavaged Env38 proteins (SU and TM subunits) with anti-Env38 antibody. When detected with digital gel image system (Tanon 4500), the membrane incubated with anti-GAPDH antibody was exposed for 5s; whereas, the membrane incubated with anti-flag antibody was exposed for 60s, due to the differential requirements of the optimum exposure time for GAPDH protein and flag tag. As a result, the edge contours of the membrane for GAPDH blot were not clearly displayed (as shown in figures B below). Similarly, the Furin A or Furin B cleavaged Env38 proteins (SU and TM subunits) examined with anti-Env38 antibody in Fig. 2D were also detected in the same samples/blots as shown as follows.

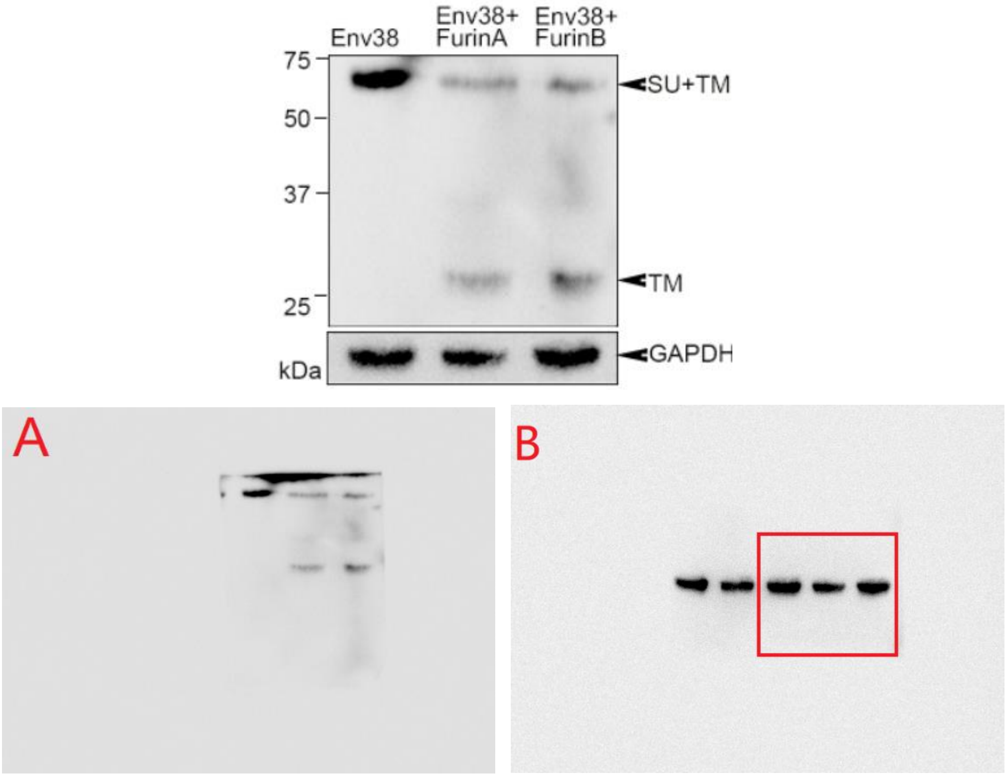

Western blot analysis for the expression and FurinA/FurinB cleavage of Env38 protein. Coexpression of Env38 with FurinA/FurinB in HEK293T cells resulted in cleavage of Env38 into TM subunit with an expected molecular weight of 25 kDa as detected with anti-flag antibody. Fig A and Fig B are the original images of Env38 proteins (A) and GAPDH (B) before and after FurinA/FurinB treatment.

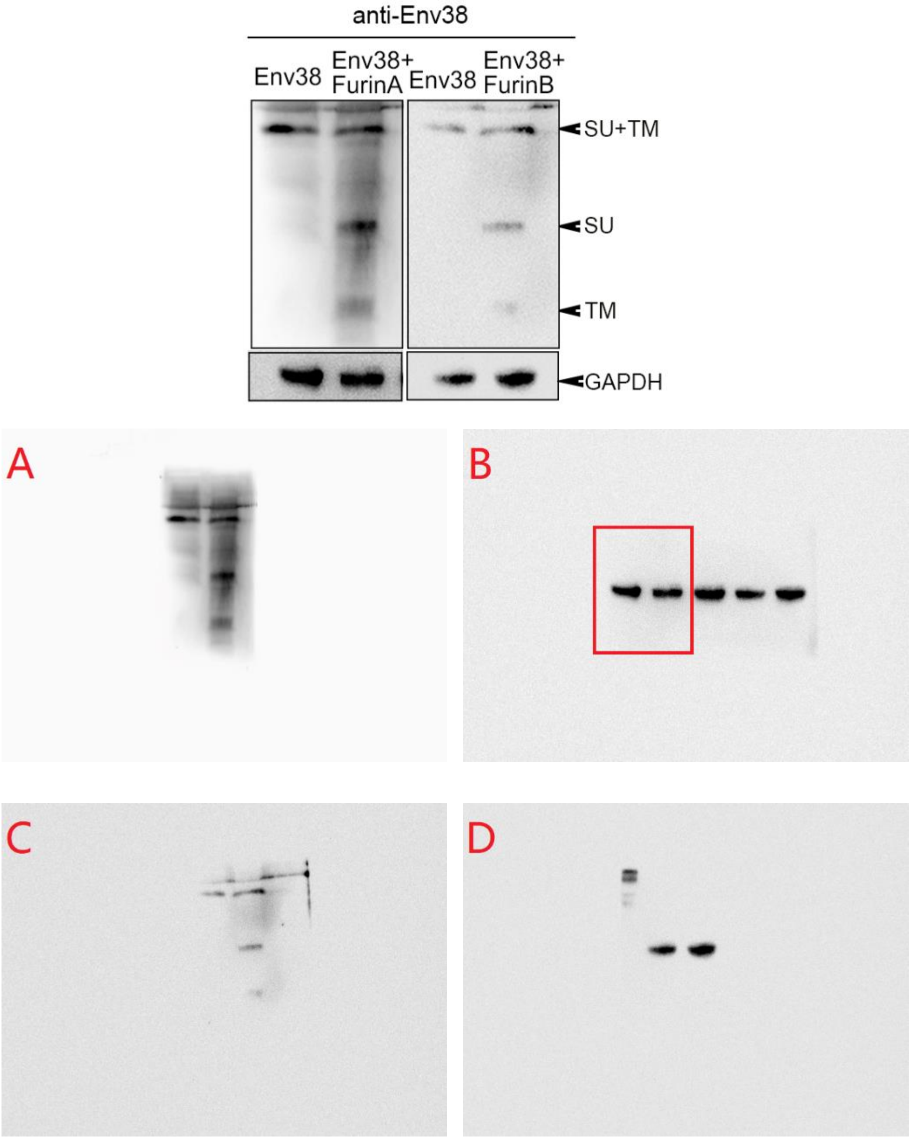

Western blot analysis for the expression and FurinA/FurinB cleavage of Env38 protein. Coexpression of Env38 with FurinA/FurinB in HEK293T cells resulted in cleavage of Env38 into SU and TM subunits with expected molecular weights of 35 kDa and 25 kDa as detected with anti-Env38 antibody. Fig A and Fig B are the original images of Env38 proteins (A) and GAPDH (B) before and after FurinA treatment. Fig C and Fig D are the original images of Env38 proteins (C) and GAPDH (D) before and after FurinB treatment.

Overall, however, the manuscript has significantly improved and the authors' main conclusion are supported by the data shown.

Reviewer #3: N/A

Part II – Major Issues: Key Experiments Required for Acceptance
Please use this section to detail the key new experiments or modifications of existing experiments that should be absolutely required to validate study conclusions.

Generally, there should be no more than 3 such required experiments or major modifications for a "Major Revision" recommendation. If more than 3 experiments are necessary to validate the study conclusions, then you are encouraged to recommend "Reject".

Reviewer #1: (No Response)

Reviewer #2: None

Reviewer #3: N/A

Part III – Minor Issues: Editorial and Data Presentation Modifications
Please use this section for editorial suggestions as well as relatively minor modifications of existing data that would enhance clarity.

Reviewer #1: (No Response)

Reviewer #2: As mentioned above, I feel that the authors need to change their interpretation of Fig. 9A, given the differences in survival between WT and KO animals in the absence of immunization.

Response: As per reviewers' suggestion, we have changed the interpretation of Fig. 9A in the revised manuscript (lines 403-407).

Reviewer #3: the authors have addressed my comments.

PLOS authors have the option to publish the peer review history of their article (what does this mean?). If published, this will include your full peer review and any attached files.

If you choose "no", your identity will remain anonymous but your review may still be made public.

Do you want your identity to be public for this peer review? For information about this choice, including consent withdrawal, please see our Privacy Policy.

Reviewer #1: **Yes:** Yes

Reviewer #2: No

Reviewer #3: No
