# Supplementary material for: Essential role of an ERV-derived Env38 protein in adaptive humoral immunity against an exogenous SVCV infection in a zebrafish model
Source: PLoS Pathog. 2023 Apr 4;19(4):e1011222. doi: 10.1371/journal.ppat.1011222 (PMC10072453; doi:10.1371/journal.ppat.1011222)
Supplement: Attachment — Submitted filename: Point-by-point response.docx [file ppat.1011222.s013.docx]

**Point by point Responses to editors’ comments**

We gratefully appreciate the editors for their time and insightful comments, which have enabled us to improve the quality of our work. Each suggested revision and comment have been accurately incorporated and considered. Our responses and revisions are indicated beneath the comments point by point.

Editor:

Because the edges of the blots are not detectable on the scanned images that you have provided in support of Figure 2, we have not been able to reach a definitive conclusion regarding data integrity. We thus request that you generate a new version of figure 2, from a replicate experiment, and provide the underlying blots to allow for proper inspection.

Response: Thanks for the comments. We have generated a new version of Figure 2, in which the Fig.2A, Fig.2B, Fig.2D, Fig.2E and Fig.2F are generated from replicate experiments, in the revised manuscript, and provided the underlying blots with detectable edges to allow for proper inspection in the additional information needed for PPATHOGENS-D-22-00798 and research data. All changes made to this revised version are highlighted in yellow for your easy review.

Fig.2A Western blot analysis for the expression of Env38 protein with mouse anti-His Ab (1:5,000). Fig. A is the original image of Env38 proteins expressed in prokaryotic E. coli cells. Fig. B is the original image of Env38 proteins expressed in eukaryotic Sf9 cells.

Fig.2B Western blot analysis for N-glycosylation of Env38 protein with treatment of PNGase F and tunicamycin, in which the Env38 protein derived from Sf9 cells was treated with PNGase F (500 U/ml), and the Env38-expressing HEK293T cells transfected with pcDNA3.1-Flag-Env38-LTR (0.6 µg/ml) was treated with tunicamycin (5 µg/ml). The Env38 protein was separated by 12% SDS-PAGE. Env38 protein derived from Sf9 cells without digestion and HEK293T cells without treatment were served as controls. GAPDH was used as a loading control. Fig A is the original image of Env38 protein before and after PNGase F digestion. Fig B is the original image of Env38 protein before and after tunicamycin treatment. Fig C is the original image of GAPDH. Env38 and GAPDH were detected on the same membrane. Because of the large differences in protein expression productivity and molecular weight which would interfere, we detected GAPDH and Env38 respectively. First, we detected he deglycosylation of Env38 protein with anti-flag antibody, and then striped the anti-flag antibody with Stripping Buffer (Thermo Scientific) on the same membrane. Next, the same membrane was re-blocked and incubated with anti-GAPDH antibody to detect GAPDH.

Fig. 2D Western blot analysis for the expression and FurinA/FurinB cleavage of Env38 protein. Coexpression of Env38 with FurinA/FurinB in HEK293T cells resulted in cleavage of Env38 into TM subunit with an expected molecular weight of 25 kDa as detected with anti-flag Ab. Fig A is the original image of Env38 protein before and after FurinA/FurinB treatment detected with anti-Flag. Fig B is the original image of GAPDH. Fig C is the original image of Env38 protein before and after FurinA treatment detected with anti-Env38. Fig. D is the original image of GAPDH. Fig. E is the original image of Env38 protein before and after FurinB treatment detected with anti-Env38. Fig. F is the original image of GAPDH. Env38 and GAPDH were detected on the same membrane. Because of the large differences in protein expression productivity and molecular weight which would interfere, we detected GAPDH and Env38 respectively. First, we detected Env38 protein with anti-flag/anti-Env38 antibody, and then striped the anti-flag/anti-Env38 antibody with Stripping Buffer (Thermo Scientific) on the same membrane. Next, the same membrane was re-blocked and incubated with a nti-GAPDH antibody to detect GAPDH.

Fig.2E Western blot analysis for the expression of FurinA and FurinB proteins in HEK293Ts cell. The right is the original image of the expression of FurinA and FurinB proteins in HEK293Ts cell.

Fig.2F Western blot analysis for the reactivity and specificity of mouse anti-Env38 polyclonal Ab. Fig. A is the original image of natural Env38 proteins derived from spleen, head kidney combined with anti-Env38 Ab. Fig. B is the original image of natural Env38 proteins derived from leukocytes combined with anti-Env38 Ab. Fig. C is the original image of recombinant Env38 proteins from HEK293T cells combined with anti-Env38 Ab. Fig. D is the original image of recombinant Env38 proteins from E. coli cells combined with anti-Env38 Ab.
